# Right-to-left shunt and transcranial Doppler as a diagnostic tool: when and how to run it. Position statement by the Italian society of neurosonology and cerebral haemodynamics

**DOI:** 10.3389/fneur.2025.1668891

**Published:** 2025-12-10

**Authors:** Giuseppe Miceli, Sara Trapani, Antonella Cramaro, Rita Bella, Mariagiovanna Cantone, Paolo Limoni, Marinella Marinoni, Chiara Mozzini, Andreaserena Recchia, Stefano Ricci, Federica Secone, Rossana Tassi

**Affiliations:** 1 Department of Health Promotion, Mother and Child Care, Internal Medicine and Medical Specialties (ProMISE), Palermo University, Palermo, Italy; 2 Neurosonology–SODc Neurophysiopathology, University Hospital Careggi, Florence, Italy; 3 Department of Medical and Surgical Sciences and Advanced Technologies “G. F. Ingrassia”, University of Catania, Catania, Italy; 4 Neurology Unit, Policlinico University Hospital “G. Rodolico-San Marco”, Catania, Italy; 5 Neurosonology at Hyperbaric Centre in Ravenna, Bologna, Italy; 6 Independent Researcher, Florence, Italy; 7 Department of Medicine ASST Mantova, Carlo Poma Hospital, Mantova, Italy; 8 Fondazione IRCCS Casa Sollievo Della Sofferenza San Giovanni Rotondo, Foggia, Italy; 9 ISA-AII Scientific Committee, Città di Castello, Italy; 10 UOC Neurology AST 3 PO Macerata, Macerata, Italy; 11 Stroke Unit, Department of Emergency Urgency, Azienda Ospedaliera Universitaria Senese, Siena, Italy

**Keywords:** transcranial Doppler, patent foramen ovale, right-to-left shunt, stroke, microembolic signals

## Abstract

Patent foramen ovale (PFO) represents a frequent congenital cardiac anomaly and the most common cause of right-to-left shunt (RLS), with relevant clinical implications in cryptogenic ischemic stroke among young adults. Accurate and standardized diagnostic strategies are essential to identify clinically significant shunts and guide therapeutic decisions, particularly regarding PFO closure. Under the auspices of the Italian Society of Neurosonology and Cerebral Haemodynamics (SINSEC), this position statement provides evidence-based recommendations on the diagnostic use of contrast-enhanced transcranial Doppler (c-TCD) for RLS detection. A structured literature review was conducted according to PRISMA 2020 methodology to ensure methodological transparency and rigor. The search covered PubMed, Scopus, and Web of Science databases for studies published between January 1990 and February 2025, using predefined keywords related to RLS, PFO, c-TCD, diagnostic accuracy, and procedural protocols. Eligible studies were screened and critically appraised to synthesize current evidence on diagnostic applications, methodological variability, and clinical utility of c-TCD. Subsequently, a Delphi consensus process was undertaken among representatives from 11 neurosonology centers with recognized expertise in cerebrovascular diagnostics. Three iterative rounds were conducted to reach agreement on key aspects of the c-TCD protocol, including patient preparation, contrast agent administration, monitoring settings, and shunt grading criteria. Draft recommendations were developed, revised collectively, and approved through a final plenary consensus meeting. The resulting position statement defines a standardized protocol for the execution, interpretation, and reporting of c-TCD in RLS diagnosis. Adoption of these consensus-based recommendations aims to enhance diagnostic accuracy, improve inter-center consistency, and support the broader clinical and research integration of c-TCD in cerebrovascular diagnostics.

## 1 Introduction

Transcranial Doppler (TCD) ultrasonography represents a cornerstone in the non-invasive assessment of cerebral hemodynamics, allowing real-time evaluation of intracranial blood flow velocity and detection of embolic or shunting phenomena. Its high temporal resolution, portability, and safety profile have made it an indispensable tool in both clinical practice and cerebrovascular research (1). The addition of an ultrasound contrast agent, contrast-enhanced transcranial Doppler (c-TCD), has expanded its diagnostic capabilities, particularly for the identification of right-to-left shunts (RLS).

Among the various causes of RLS, the patent foramen ovale (PFO) holds particular clinical relevance. PFO is a congenital cardiac abnormality resulting from the incomplete postnatal closure of the interatrial communication, persisting in approximately 25–30% of the adult population and accounting for up to 95% of RLS cases (2). Its frequent association with cryptogenic ischemic stroke in young adults has led to increasing recognition of its potential pathogenic role. Given that about 10% of all strokes occur in individuals aged 18–50 years, and that this incidence is rising partly due to the growing prevalence of vascular risk factors (3, 4), accurate identification of clinically significant PFO is essential for selecting appropriate candidates for closure and preventing stroke recurrence.

Several imaging modalities can be employed for RLS detection, including contrast-enhanced transesophageal echocardiography (c-TEE), contrast-enhanced transthoracic echocardiography (c-TTE), and c-TCD (5–7). While c-TEE remains the reference standard for structural characterization of the interatrial septum, its semi-invasive nature limits its use as a screening tool. Conversely, c-TCD and c-TTE are non-invasive, widely accessible, and well-tolerated, rendering them suitable for initial evaluation (5). Among these, c-TCD is often regarded as the preferred first-line method for detecting functional shunts due to its high sensitivity, repeatability, and ability to quantify shunt magnitude in real time.

Despite its advantages, the clinical implementation of c-TCD remains highly heterogeneous across centers. Variations exist in technical parameters, contrast preparation and injection protocols, patient maneuvers, and interpretation criteria, leading to differences in diagnostic accuracy and inter-operator reproducibility (8). The absence of a unified, evidence-based protocol hinders both the reliability of single-center results and the comparability of multicenter studies. Establishing standardized operational and interpretative procedures is therefore crucial to ensure methodological consistency, improve diagnostic confidence, and facilitate the broader integration of c-TCD into routine cerebrovascular assessment and multicenter research.

This position paper aims to define a comprehensive, standardized protocol for the execution, interpretation, and reporting of c-TCD in the diagnosis of right-to-left shunts. By consolidating expert consensus and current best practices, this document seeks to enhance diagnostic precision, promote inter-center uniformity, and support the optimal clinical and research use of contrast-enhanced transcranial Doppler in the evaluation of PFO-related and other RLS conditions.

## 2 Methodology

**Figure 1:**
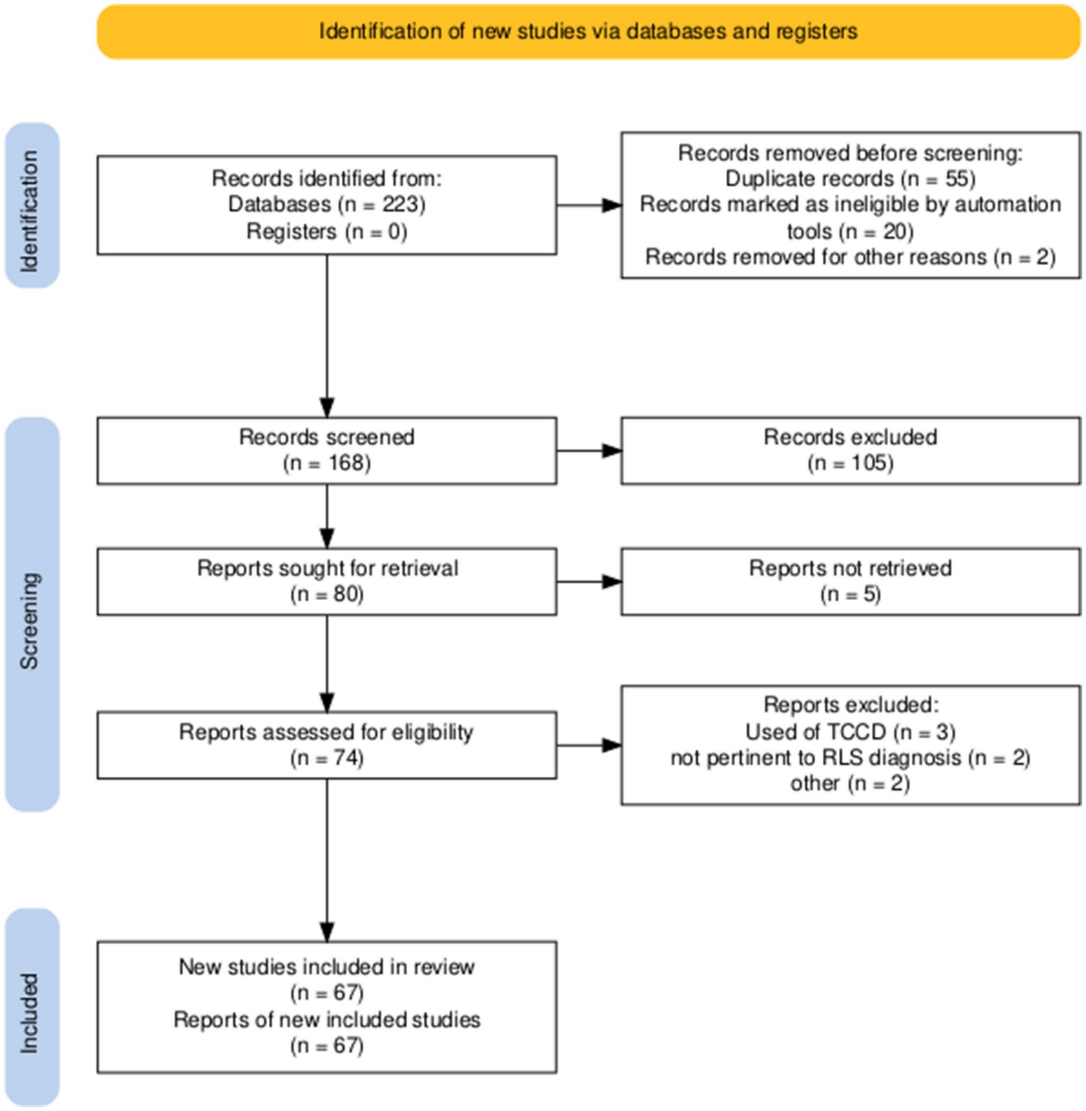
Research flow diagram according to PRISMA methodology.

This position statement was developed under the auspices of the Italian Society of Neurosonology and Cerebral Haemodynamics (SINSEC) to provide evidence-based guidance on the diagnostic use and methodology of contrast-enhanced c-TCD for detecting right-to-left shunt (RLS). A structured literature review was conducted following the Preferred Reporting Items for Systematic Reviews and Meta-Analyses (PRISMA) methodology (9) to ensure transparency, reproducibility, and rigor in the identification and synthesis of the available evidence (Figure 1).

A comprehensive search was conducted in the PubMed, Scopus, and Web of Science databases, encompassing publications from January 1990 to February 2025. The following keywords and their combinations were used: “right-to-left shunt,” “patent foramen ovale,” “transcranial Doppler,” “contrast-enhanced ultrasound,” “Valsalva maneuver,” and “diagnostic accuracy.” Only articles published in English were considered. Particularly, the literature search was conducted in the PubMed database using the following predefined search string:

((“right-to-left shunt”[Title/Abstract] OR “RLS”[Title/Abstract] OR “patent foramen ovale”[Title/Abstract] OR “PFO”[Title/Abstract] OR “pulmonary arteriovenous malformation”[Title/Abstract] OR “PAVM”[Title/Abstract] OR “Eisenmenger syndrome”[Title/Abstract] OR “migraine”[Title/Abstract] OR “platypnea-orthodeoxia syndrome”[Title/Abstract] OR “diving”[Title/Abstract] OR “diver”[Title/Abstract] OR “decompression illness”[Title/Abstract])) AND (“transcranial Doppler”[Title/Abstract] OR “TCD”[Title/Abstract] OR “contrast-enhanced transcranial Doppler”[Title/Abstract] OR “c-TCD”[Title/Abstract] OR “transcranial color-coded Doppler”[Title/Abstract] OR “TCCD”[Title/Abstract]) AND (“diagnostic accuracy”[Title/Abstract] OR “sensitivity”[Title/Abstract] OR “specificity”[Title/Abstract] OR “protocol”[Title/Abstract] OR “standardization”[Title/Abstract] OR “Valsalva maneuver”[Title/Abstract]) AND (“humans”[MeSH Terms] OR “infant”[MeSH Terms] OR “child”[MeSH Terms] OR “adolescent”[MeSH Terms] OR “pregnancy”[MeSH Terms]) AND (“1990/01/01”[Date - Publication]: “2025/02/28”[Date - Publication]) AND (English[lang]). This search strategy was designed to identify studies addressing the diagnostic application of c-TCD and related ultrasound techniques for detecting RLS in various clinical contexts, including PFO, pulmonary arteriovenous malformations, Eisenmenger syndrome, migraine, dive-related decompression illness, and platypnea-orthodeoxia syndrome. Studies involving adult, pediatric, and pregnant populations were included. The search was restricted to articles published in English between January 1990 and February 2025 and conducted on human subjects. Reference lists of relevant reviews, meta-analyses, and consensus papers were also screened to identify additional studies. Eligible articles included those involving human participants and reporting data on diagnostic protocols, methodological variations, sensitivity and specificity, or clinical applicability of c-TCD in RLS detection. All retrieved evidence was independently reviewed by a panel of experts in neurosonology and cerebrovascular diagnostics designated by SINSEC. To define the recommended protocol for c-TCD execution, a Delphi consensus process (10) was conducted among representatives from 11 centers with proven expertise in neurosonology and cerebrovascular diagnostics. The characteristics of these centers are summarized in Supplementary Table 1. The Delphi process consisted of three iterative rounds that aimed at achieving consensus on key procedural aspects, including patient preparation, contrast agent preparation and injection, monitoring settings, and criteria for shunt grading. Each section of the manuscript was drafted by subgroups of the expert panel, critically reviewed by all contributors, and finally approved through a plenary consensus meeting. Recommendations were formulated based on the strength of available evidence and expert agreement, in alignment with current international standards for position statements.

## 3 Right-to-left shunt: physiology and associated pathological conditions

RLS is an abnormal shunting of blood through the cardiovascular and pulmonary systems, bypassing the pulmonary capillary circulation.

The most common cause of RLS is the presence of a PFO; however, other causes cannot be entirely ruled out, such as Pulmonary Arteriovenous Malformation and Eisenmenger Syndrome.

### 3.1 Patent foramen ovale

The development of the bilateral atria and the *foramen ovale* begins around the fourth week of embryogenesis. The *septum primum* initially divides the primitive atrium into right and left chambers, leaving an opening called the *ostium primum*, which later closes as the *ostium secundum* forms through a process of programmed cell death. By the 12th week, the *septum secundum* develops, partially covering the *foramen secundum* and creating the *foramen ovale*, a crucial passage for oxygenated blood from the placenta to bypass the non-functioning fetal lungs (11).

At birth, the drop in pulmonary pressure leads to an increase in left atrial pressure, pushing the septum primum against the septum secundum, sealing the foramen ovale and forming the fossa ovale. In approximately 75–80% of individuals, this closure is permanent. However, in 20–25% of people, the septa do not fully fuse, leaving a PFO. In a certain percentage of cases, RLS is associated with an atrial septal aneurysm (ASA), defined as a redundancy of the septum primum with a displacement of more than 10 mm from the mid-septum into either atrial chamber or a total excursion of 15 mm between the right and left atria during a cardiorespiratory cycle (12). ASA is present in 2–3% of the population (13).

The existence of a PFO after birth is associated with various medical conditions throughout different age spans (12).

The persistence of PFO is associated with potential complications such as paradoxical embolism and, in rare cases, infective endocarditis and thrombosis, all of which increase the risk of stroke. Additionally, PFO may coexist with other anomalies, such as Chiari’s network and Eustachian valve, remnant embryonic structures that might influence the convergence of the blood jet coming from the inferior vena cava toward the fossa ovalis.

Even though not a true atrial septal defect, PFO is considered a congenital malformation from a pathophysiological perspective.

### 3.2 Pulmonary arteriovenous malformation

Another condition responsible for RLS is pulmonary arteriovenous malformations (PAVMs). PAVMs are rare pulmonary vascular malformations, more common in females than males, and are often diagnosed in adulthood, although they may develop during childhood. PAVMs create a direct connection between the pulmonary artery and vein, bypassing the normal pulmonary capillary bed, which is responsible for gas exchange and filtration (14).

Although the etiology of PAVMs is not fully understood, 80–90% are congenital and occur either as isolated abnormalities or as part of hereditary hemorrhagic telangiectasia. Due to their rarity and non-specific clinical manifestations, PAVMs are not easily or routinely diagnosed. Chest X-ray, computed tomography (CT), and magnetic resonance imaging (MRI) are the primary diagnostic tools, with CT considered the *gold standard* for its superior anatomical visualization of pulmonary parenchyma and vascular structures. Many patients with PAVMs remain asymptomatic, particularly when the malformation is less than 2 mm in diameter. However, larger PAVMs (≥2 mm) or feeding arteries exceeding 3 mm may cause chest pain, cough, dyspnea, palpitations, or hemoptysis. The severity of symptoms depends on the number, size, and extent of the shunt (15). PAVMs significantly increase the risk of embolic complications due to the RLS, and neurological manifestations are common. Reports indicate that 2.4–58.7% of PAVM patients experience conditions such as ischemic stroke, transient ischemic attack (TIA), cerebral abscess, migraine, or hemorrhage. Among these, ischemic stroke and brain abscesses are the most frequently observed. Stroke is the most prevalent neurological complication of PAVMs, as paradoxical embolism allows unfiltered microemboli to bypass the pulmonary capillary network and enter the cerebral circulation. This mechanism is widely recognized as a potential cause of acute ischemic stroke, although PAVMs account for only a small fraction of ischemic stroke admissions (0.02%) (14).

### 3.3 Eisenmenger syndrome

Eisenmenger Syndrome (ES) is a complex condition that arises from certain congenital heart defects, such as a ventricular septal defect, atrial septal defect, or patent ductus arteriosus. These defects initially cause RLS, leading to increased blood flow to the lungs. Over time, this increased flow results in elevated pulmonary vascular resistance and pulmonary hypertension. As the pulmonary pressure surpasses systemic pressure, the shunt reverses to an RLS, causing cyanosis due to mixing of oxygenated and deoxygenated blood (16, 17).

.In regions with advanced medical care, early detection and surgical correction of congenital heart defects have significantly reduced the incidence of ES. The prevalence of pulmonary arterial hypertension within the adult congenital heart defects population remains uncertain. In 2014, data suggest a prevalence of over 10% for any pulmonary arterial hypertension, but likely much lower for ES (18). European data suggest that the prevalence of ES in significant congenital heart disease varied between 1 and 5.6% (16, 17).

ES is characterized by chronic hypoxemia and multisystem involvement, including secondary erythrocytosis (often accompanied by iron deficiency), an increased risk of thrombosis and bleeding, a high burden of arrhythmias, susceptibility to infections, and progressive heart failure (HF), necessitating multidisciplinary management in specialized centers (19).

## 4 Clinical conditions warranting investigations for PFO

### 4.1 PFO and stroke

One-third of ischaemic strokes are considered to be cryptogenic, without identifiable cause, with a significant prevalence among younger patients (20). About 16% of cryptogenic strokes are further identified as embolic strokes of undetermined source (21), and in a recent paper, Sposato and Coauthors estimate that 25% of these patients have a PFO (22), which signifies that a PFO may be implicated in up to 4% of all patients with ischemic stroke. On this basis, a shift in nomenclature has been proposed to reflect this probable causality, with the term PFO-associated stroke (23). The cause of the stroke in these patients is called “Paradoxical embolism.” This includes a thrombus straddling a PFO, a thrombus that develops in the systemic venous circulation, bypassing lung filtration and crosses directly into the cerebral arterial circulation, and lastly, recent investigations using high-resolution optical coherence tomography have found a high frequency of *in situ* thrombi in stroke and migraine patients, suggesting that thrombi may develop within the PFO itself (24).

Since not all PFOs confer the same stroke risk, it is essential to identify those with a higher risk of stroke recurrence and who may derive more benefits from closure. The size of PFO (moderate/large) with the concomitant presence of ASA confers a high risk of recurrence, together with other cardiac morphological features such as the Eustachian valve and Chiari’s network (15).

Two risk stratification tools have been proposed to assess the likelihood that a cryptogenic stroke may be attributable to a PFO. The first, the Risk of Paradoxical Embolism (RoPE) score, is based solely on clinical parameters (25), whereas the second, the PFO-Associated Stroke Causal Likelihood (PASCAL) classification, integrates both the RoPE score and anatomical characteristics of the PFO (21). The RoPE and PASCAL scoring systems may be utilized to identify patients most likely to benefit from transcatheter PFO closure (22, 23). Scientific evidence suggests that individuals with both a large right-to-left interatrial shunt and the presence of an atrial septal aneurysm get the most therapeutic benefit from closure (22).

In clinical practice, PFO closure is performed in combination with antiplatelet therapy for carefully selected patients aged 18–60 years who have experienced a cryptogenic stroke in the absence of an identifiable alternative etiology.

### 4.2 PFO and stroke during pregnancy

The prevalence of congenital heart disease in women of childbearing age is increasing due to childhood interventions and improved methods of detection. The presence of pregnancy as an isolated factor is generally not considered a sufficient indication for PFO closure. But in women with a history of ischemic stroke and a known PFO, percutaneous device closure should be considered before conception, as available evidence suggests it may offer superior protection compared to medical therapy alone (26). The presence of a PFO confers a theoretical risk of paradoxical embolism, which becomes particularly relevant during pregnancy due to the progressive hypercoagulable state.

The risk of cryptogenic stroke associated with PFO is increased during pregnancy, particularly in the presence of venous thromboembolism (VTE), and may extend into the postpartum period (26). These strokes tend to occur in the first and second trimesters, and are more frequently associated with multiple gestations, anatomical factors such as an interatrial septal aneurysm and large RLS, or coexisting hypercoagulable conditions (27, 28). During labor, prolonged Valsalva maneuvers (VM) may transiently increase right-to-left shunting, potentially facilitating paradoxical embolization (27).

In the acute setting, particularly during the first trimester, treatment may include low-dose aspirin and low-molecular-weight heparin (28). If anticoagulation is contraindicated or if recurrent embolic events occur despite medical therapy, transcatheter PFO closure may be indicated during pregnancy. The procedure can be performed using intracardiac echocardiographic guidance, minimizing maternal and fetal radiation exposure (26).

Echocardiographic assessment is valuable both for differentiating a PFO from an atrial septal defect (ASD)—the latter typically associated with a larger shunt and right heart dilation—and for evaluating device positioning and function.

Although direct evidence on the safety of bubble contrast TCD during pregnancy is limited, no theoretical rationale suggests that the use of agitated saline poses harm. Therefore, this imaging modality is generally considered safe and should not be withheld when clinically indicated in pregnant patients (28). However, at present, there is no evidence to demonstrate the safety or potential harm to the fetus of administering agitated saline for diagnostic purposes during pregnancy. No studies, not even on animals, have been conducted on this subject. Nonetheless, in clinical practice, given the importance of diagnosing stroke secondary to PFO during pregnancy, many gynecologists recommend performing the test in the event of acute stroke. It is therefore reasonable to hope for an evaluation by a multidisciplinary team involving gynecologists, neurologists, and operators who deal with the diagnosis of PFO that can identify patients who need to undergo c-TCD.

### 4.3 PFO and stroke in pediatric patients

Despite its low incidence, ischemic stroke can also affect the pediatric population, and its causes often remain unknown. Although certain risk factors may be present, they are not necessarily causative. These factors may contribute to disease pathogenesis only following a triggering event, such as an acute infection or anemia (29). Like in the adult population, RLS may result in embolization in children, though a definitive causative link between PFO and ischemic stroke has not been established. Identifying PFO as the cause of AIS is generally based on exclusion after thoroughly ruling out other etiologies, and PFO closure should be considered on an individual basis (30).

c-TEE, c-TTE, and c-TCD can serve as diagnostic tools as well as in adults even if in the pediatric population, deep sedation is needed for TEE, and the Valsalva maneuver cannot be performed by the patient. Moreover, children generally have a superior acoustic window for TTE compared to adults. Currently, there are no available studies on c-TCD in children; however, its high sensitivity and specificity suggest that it could be an effective screening tool for RLS in children capable of performing the Valsalva maneuver.

Conclusions regarding PFO treatment in children are not yet available, as the American Heart Association consensus statement does not provide a recommendation for PFO closure to prevent recurrent strokes in the pediatric population due to insufficient evidence (31).

Contrast TCD appears to be the best diagnostic option for detecting RLS in children; however, the consensus conference by Zauss and Zanette (8) standardized the diagnostic procedures for adult patients and did not provide recommendations for the pediatric population.

The lack of a standardized procedure produces ambiguities, and many questions remain unresolved:

- What gage needle is adequate? Is it different according to weight and assumed venous caliber?
- What is the adequate dose of contrast solution? In what proportion is saline/air?
- In order to interpret results, can we use the same grading?

### 4.4 PFO and migraine

While evidences suggest an association between PFO and migraine, particularly migraine with aura, the clinical utility of PFO research in this context remains controversial (32, 33). While the pathophysiological mechanisms, ranging from bypass of pulmonary filtration, microembolic phenomena, alterations in serotonin metabolism, to inflammatory responses, offer plausible explanations for this relationship, definitive causal links remain to be fully established. Although PFO closure has shown promising results in reducing migraine symptoms in some observational studies, the mixed outcomes of randomized controlled trials have precluded its routine use as a migraine prophylactic measure (34). Ongoing research is essential to elucidate the underlying mechanisms and to identify patient subgroups who might benefit from targeted interventional strategies. However, actual professional guidelines do not currently recommend routine screening for PFO in migraine patients due to insufficient evidence that PFO closure or detection significantly impacts migraine management or outcomes (35). In summary, while c-TCD can be used to detect PFO, its routine use in the diagnostic work-up of migraine is not supported by current clinical guidelines.

### 4.5 PFO and dive activities

Decompression sickness (DCS) involves one or more organs and results from the expansion and systemic distribution of gas bubbles before they are reabsorbed. It occurs following a transition from a high-pressure environment to a lower-pressure one, and can thus arise during underwater diving, as well as in settings such as hyperbaric therapy or space travel (36).

Symptoms are caused by tissue supersaturation with dissolved gasses, which may exceed the filtering capacity of the pulmonary circulation, or by the entry of bubbles into the arterial system, either through RLS or secondary to pulmonary barotrauma.

The estimated incidence of DCS varies widely, ranging from 0.9 to 35.3 cases per 10,000 person-dives in recreational, commercial, instruction-led, and military diving. In contrast, experienced divers show a much lower incidence of approximately 0.324 per 10,000 person-dives (36).

Although DCS remains relatively rare when scuba diving is properly conducted, available evidence supports a significant association between the occurrence of DCS and the presence of a large PFO (12). A recent meta-analysis examining the relationship between RLS and DCS, as well as the potential link between RLS and silent brain lesions in scuba divers without a prior history of DCS, yielded the following findings: the presence of RLS was found to significantly elevate the risk of neurological decompression sickness (NDCS), particularly in individuals with high-grade shunts. No statistically significant association was observed between RLS and asymptomatic brain lesions on MRI in otherwise healthy divers. While RLS is recognized as a risk factor for NDCS, it is essential to note that not all divers with RLS experience this complication, nor can all cases of NDCS be attributed to the presence of RLS (36).

### 4.6 RLS and other pathologies

Platypnea-orthodeoxia syndrome (POS) is characterized by systemic oxygen desaturation that occurs in the upright position and resolves when supine, and is typically secondary to an RLS. In most cases, POS is caused by a PFO, although it can also result from PAVMs. Although considered uncommon, previously published data have shown that POS accounts for over 5% of all PFO closure procedures performed over 15 years (12).

Interestingly, POS most often affects patients with a mean age of around 65 years, which contrasts with the congenital nature of PFO, a condition present from birth, highlighting that the syndrome may become clinically manifest only later in life due to anatomical or functional changes.

## 5 PFO: what is the best diagnostic tool? International guidelines and recommendations

The precise identification of PFO-associated right-to-left shunting and detailed characterization of the PFO anatomy in patients with cryptogenic stroke are essential for risk assessment and treatment planning. The primary diagnostic tools employed for detecting and evaluating RLS include c-TCD, c-TTE, and c-TEE.

- c-TEE enables detailed visualization of the interatrial septum and facilitates accurate assessment of the size and morphology of a PFO. However, due to its semi-invasive nature, performing an adequate VM during the procedure can be challenging, which may compromise diagnostic sensitivity and reduce inter-study reproducibility. Nonetheless, because of its ability to characterize directly the anatomical features of the PFO, c-TEE is frequently regarded as the preferred imaging modality for intracardiac evaluation and has served as the cornerstone in subsequent diagnostic protocols (37).
- c-TTE in a recent meta-analysis of 35 studies involving 4,209 patients, an excellent specificity and moderate sensitivity of c-TTE emerged for RLS diagnosis compared to c-TEE. The Author suggested that it might serve as an initial screening modality for selected patients with a high likelihood of having RLS and for indications for treatment (38).
- c-TCD represents a non-invasive and well-tolerated modality for the detection of RLS, enabling standardized performance of the VM. Nevertheless, its diagnostic utility is limited by the requirement for an adequate temporal bone window. Although it cannot determine the precise anatomical location of RLS, thus limiting its specificity in differentiating cardiac from extracardiac sources, it remains a valuable screening tool, particularly for detecting PFO, the most common cause of RLS.

The literature provides several data regarding the sensitivity and specificity of the three diagnostic techniques. In the 2016 meta-analysis by Katsanos et al., which included 35 studies published between 1991 and 2011, the diagnostic superiority of c-TCD over c-TTE for detecting RLS was highlighted. c-TCD was found to be more sensitive but less specific than c-TTE in identifying PFO in patients with cryptogenic stroke or TIA. Notably, the overall diagnostic accuracy of c-TCD was nearly comparable to that of c-TEE, making it a reasonable initial screening method for RLS detection in patients with cryptogenic cerebral embolism (39).

More recent reports demonstrate that c-TTE has relatively low sensitivity, ranging from 30 to 80%, and fails to detect a significant number of PFOs, including high-risk cases (38, 39). c-TEE, while more sensitive than c-TTE, is often not easily feasible in the subacute setting and has demonstrated lower sensitivity compared with c-TCD for detecting RLS (40, 41). Recent findings in a 2024 review published in Stroke indicate that the accuracy of c-TCD varies depending on the center, protocol, and diagnostic criteria used, with sensitivity ranging from 70 to 100% and specificity exceeding 95% when compared with c-TEE, as per the American Academy of Neurology (27). Interestingly, the sensitivity of c-TCD to detect RLS, including those caused by PFO, appears to be higher than that of c-TEE, as demonstrated in multiple studies (40–43). This raises questions about whether c-TEE is truly the nonsurgical gold standard for RLS detection. For example, power motion TCD has proven more sensitive than c-TEE in detecting significant RLS when compared with anatomic findings from catheterization (44). The superior sensitivity of c-TCD may be partially attributed to the challenges in performing an effective VM during c-TEE. Regarding the c-TCD procedure, unilateral MCA insonation did not lower the diagnostic accuracy statistics compared to bilateral MCA monitoring (39).

On this basis, current guidelines recommend in patients with cryptogenic stroke, the optimal diagnostic strategy for identifying an RLS remains uncertain, largely due to the absence of a universally accepted reference standard. Consequently, an evidence-based consensus on the preferred modality cannot be established at this time. In light of this limitation, the implementation of institution-specific diagnostic pathways that incorporate the available imaging techniques, c-TCD, c-TTE, and c-TEE, is recommended. In cases where c-TTE and/or c-TEE yield non-diagnostic or equivocal results, c-TCD should be considered as an alternative approach. In case of patients with cryptogenic stroke, and initial evidence of a RLS, clinical guidelines recommend the use of c-TEE to further characterize the shunt and to provide detailed information on the presence, anatomical configuration, and potential pathogenic role of a PFO (3, 6, 45–47).

## 6 Transcranial Doppler in RLS

Transcranial Doppler is a non-invasive, low-cost, and repeatable technique that allows measurement of blood flow velocities in the arteries of the Circle of Willis. TCD allows the evaluation of Middle, Anterior, and Posterior Cerebral Arteries through temporal acoustic windows, as well as Vertebral and Basilar Arteries through the suboccipital window. Additionally, the Carotid Siphons can be assessed using transorbital windows. To accurately identify each artery and optimize the received signal, the following parameters are adjusted on the TCD device: depth of insonation, sample volume, pulse repetition frequency, gain, and power (48).

### 6.1 Microemboli detection by transcranial Doppler

While ultrasound typically focuses on analyzing the frequency of the returned signal, its intensity can provide indirect information about materials encountered by the beam, potentially identifying circulating emboli. The intensity depends on the difference in acoustic impedance between tissues, which correlates with density differences. For particles smaller than the ultrasound wavelength (0.77 mm for a 2-MHz transcranial Doppler probe), Rayleigh scattering governs reflection, scattering ultrasound in all directions, with some returning to the probe. Both direct reflection and Rayleigh scattering result in increased signal intensity when an embolus passes through the beam, allowing for theoretical detection of emboli based on density differences.

Air, with its very low density, creates a significant acoustic impedance difference compared to blood, making gaseous emboli particularly easy to detect (49).

In 1995, the Consensus Committee of the 9th International Cerebral Hemodynamic Symposium published the foundational Basic Identification Criteria for Doppler Microembolic Signals, which are still widely referenced today. According to these criteria:

1. A Doppler microembolic signal is transient, typically lasting less than 300 milliseconds;
2. Its amplitude is usually at least 3 decibels higher than the surrounding blood flow signal.
3. The signal is unidirectional within the Doppler velocity spectrum, providing that bidirectional Doppler equipment is used properly and the dynamic range settings are appropriate.
4. Depending on the device and the embolus velocity, the signal is accompanied by a characteristic sound on the audio output, described as a “snap,” “chirp,” or “moan” (50).

The physical principle underlying the detection of microbubbles (MB) in the contrast agent used in TCD for the diagnosis of RLS is exactly the same.

The algorithm underlying microemboli recognition software processes not only the intensity and duration of the signal but also its unidirectionality, by analyzing how the signal propagates across different insonation depths. There are at least two main detection approaches: one involves identifying emboli through the analysis of two gates positioned 5–10 mm apart, while the other is the multigate method described by Spencer et al. in 2004 (44).

**Figure 2:**
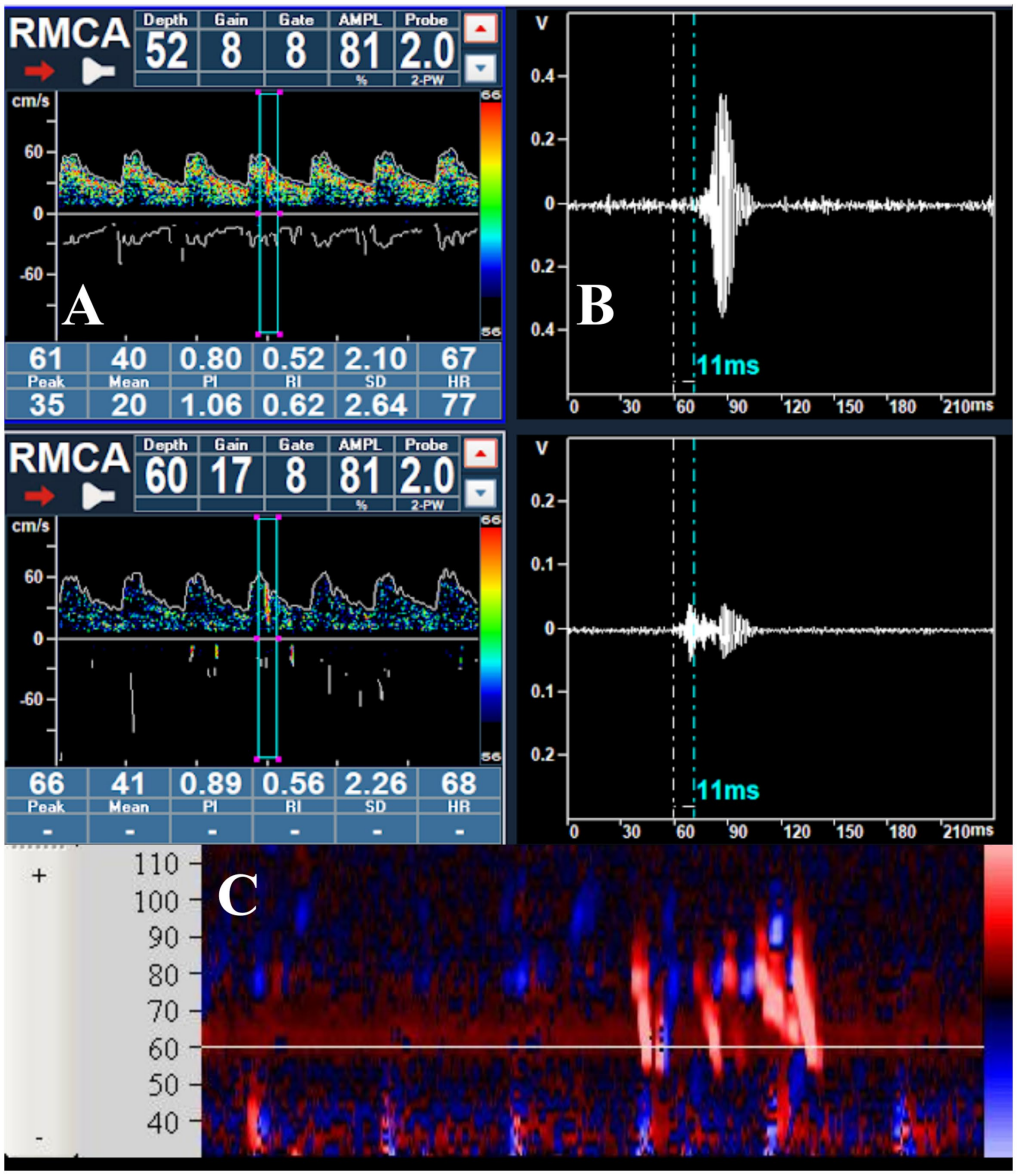
**(A)** Passage of a microbubble visualized within the Doppler spectrum during the right middle cerebral artery monitoring; **(B)** raw data of the MB, showing the delay in appearance at a depth of 52 mm; **(C)** visualization of a series of MBs in the M-mode.

All signals detected by transcranial Doppler as either microembolic events or artifacts are stored by the system and can be reviewed offline. The signal log specifies whether the event was classified as an embolus or artifact, its signal intensity, and—when bilateral monitoring is performed—the artery of origin. The operator can review each detected signal in three different modes: within the Doppler spectrum (Figure 2A), in M-mode (Figure 2C), or as raw data (Figure 2B); in addition, the acoustic output of each signal can typically be replayed for auditory verification.

Keunen et al. developed an alternative Embolus Detection System (EDS) based on the analysis of transcranial Doppler audio signals. The algorithm relies on information derived from the zero-crossing frequency of the Doppler audio waveform, while the fast Fourier transform (FFT) is employed solely as a visual aid for subsequent expert review. The classification of audio signals is performed using a multi-layer neural network, which evaluates the duration, intensity, and zero-crossing characteristics of each signal (51). To the best of our knowledge, this software has not gained widespread clinical adoption, and appears to demonstrate limited reliability when compared with human expert interpretation in the detection of emboli in patients undergoing carotid endarterectomy (52).

### 6.2 Limitation of c-TCD and comparison between c-TCD vs. c-transcranial color coded Doppler

Apart from operator and interpreter dependency, which are major concerns in all ultrasound examinations, TCD examination is also limited by the absence of temporal “windows,” which leads to unsuccessful insonation in 10 to 12% of patients older than 60 years (53, 54).

Some authors have explored the possibility of assessing the presence of RLS using the suboccipital window to monitor the vertebrobasilar circulation. When comparing c-TCD performed by monitoring the MCA with c-TCD monitoring the vertebrobasilar system, the latter showed a sensitivity of 57% for RLS at rest and 84% after the VM. In cases of medium or large RLS, both specificity and sensitivity increased to 100%. The authors concluded that c-TCD with vertebrobasilar recording is a sensitive and specific test for RLS diagnosis (55). A limitation of this test variant concerns its accuracy in detecting small shunts and those present at rest. Additionally, the number of MB detected during MCA monitoring was not compared with that observed in vertebrobasilar monitoring. Therefore, a reliable grading of shunt severity is not possible unless the shunt is significant.

Other studies have compared basilar artery monitoring with middle cerebral artery monitoring, yielding encouraging results in favor of using the transforaminal window when adequate temporal windows are unavailable, although a perfect correlation of test results during the facilitating manoeuvre has not been demonstrated (56–58). Two of these studies included a subgroup of patients who underwent TEE to confirm the findings; however, given the limited sample size, the promising results reported should be interpreted with caution and require further confirmation (57, 58). Another alternative, in cases of bilaterally inadequate temporal acoustic windows, could be monitoring the internal carotid artery. Duan et al. (59) reported favorable results when comparing MCA and carotid siphon monitoring in a relatively large and heterogeneous cohort that included healthy individuals, patients with cryptogenic stroke, and those with migraine. Other studies have proposed monitoring different segments of the carotid artery. For instance, Perren et al. (60) suggested a submandibular approach, also yielding encouraging results. However, the currently available evidence does not yet allow the definition of a standardized protocol for internal carotid artery monitoring, nor for the use of alternative arteries to the middle cerebral artery in this type of diagnostic evaluation.

Even though TCCD might seem like a potentially helpful tool for diagnosing RLS, and the few existing studies report high sensitivity and reasonable specificity, to date, there is indeed only one study comparing TCCD with contrast-enhanced (c-TCCD) and c-TEE for this diagnosis (61). As far as we know, no specific and standardized guidelines or published protocols exist for the application of this tool in diagnosis, nor are there studies comparing c-TCD with c-TCCD.

To our knowledge, no ultrasound devices currently incorporate software capable of automatically distinguishing MBs from artifacts, and that can calculate the decibel level of high-intensity transient signals (HITS). This, theoretically, makes it impossible to distinguish the MBs from artifacts.

Another theoretical limitation is that ultrasound devices do not allow for the simultaneous monitoring of two or more different sample volumes, making it impossible to demonstrate the unidirectionality of the signal. Moreover, there is no possibility of prolonged recording and real-time monitoring, which means that the assessment remains qualitative rather than quantitative. As a result, the ability to accurately distinguish between mild and moderate RLS is lost.

Furthermore, it is essential to note that with a cardiac probe, it is impossible to fix the probe to the temporal window, increasing the risk of signal loss, especially during VM. Additionally, bilateral monitoring of both middle cerebral arteries is not feasible. Recently, a case report was published in which some authors used c-TCCD for the diagnosis of RLS. In addition to pulsed wave Doppler, these authors employed a new methodology: color M-mode. They concluded that incorporating this modality increases the sensitivity of c-TCCD in RLS evaluation and that their work demonstrates that color M-mode could serve as a valuable method for screening and monitoring RLS patients, complementing other diagnostic tools (62).

There could be advantages in using TCCD for this type of investigation. In fact, TCCD devices are more widely available than TCD, and, simply by changing probes and/or presets, the same ultrasound machine could perform all three examinations: c-TCCD, contrast-enhanced c-TTE, and c-TEE; nevertheless, the above-mentioned limitations remain (Table 1).

**Table 1:** Comparison between c-TCD and c-TCCD.

Features	cc-TCD	c-TCCD
Emboli detection software	✓	✗
Bilateral simultaneous MCA monitoring	✓	✗
Probe fixing system	✓	✗
Post processing review	✓	Partially
Typical “embolic sound” detection	✓	✓

c-TCD, contrast-enhanced Transcranial Doppler; c-TCCD, contrast-enhanced Transcranial Color-Coded Doppler; MCA, Middle Cerebral Artery.

## 7 Proposed c-TCD protocol

We present below our proposed protocol for the performance of c-TCD, developed based on the results of an expert panel consultation conducted among experienced operators (Supplementary Table 1).

Materials and instrumentation: a. Transcranial Doppler with emboli detection software, b. helmet with bilateral 2 MHz probe, c. 18-gage needle, d. isotonic saline (or contrast agent), e. syringes, f. three-way stopcock.

Patient preparation:

- The patient lies in a supine position, which generally provides greater comfort and stability during the examination; however, the seated position can also be adopted (63)
- An 18-gage needle is inserted into one of the cubital veins, which are generally preferred due to their ease of identification and adequate caliber, allowing for a reliable injection of the agitated solution.
- Insonation of at least one MCA is required; nonetheless, bilateral monitoring is recommended as it increases diagnostic reliability (39). In particular, clinical conditions such as an inadequate temporal acoustic window or carotid occlusion, unilateral monitoring is mandatory. In adult patients, the signal from the middle cerebral arteries is typically detected at a depth between 45 and 60 mm, using two flat 2-MHz probes fixed to the temporal bone by means of a dedicated head frame equipped with locking mechanisms that ensure stability throughout the entire examination. To confirm the presence of adequate temporal acoustic windows, the arterial signals can be localized with a standard handheld probe before positioning the head frame. For optimal detection of microbubble passage, it is recommended to set both gain and power output as low as possible, since excessively high signal intensity may hinder the visualization of the high-intensity transient signals generated by the microbubbles within the Doppler spectrum. When available on the ultrasound system, the M-mode display should be activated, as it allows scanning across multiple depths and enables visualization of microbubble passage through the insonated segments. Furthermore, when dual or multiple sample volume visualization is available, the distance between the sample volumes should be equal to or bigger than the sample volume length itself to ensure accurate identification of HITS (Figure 2A).
- The device settings for microbubble detection must comply with Doppler microembolic signal characteristics: transient, typically lasting less than 300 milliseconds; its amplitude is usually at least 3 decibels higher than the surrounding blood flow signal, the signal is unidirectional within the Doppler velocity spectrum, and it is accompanied by a characteristic sound on the audio output. (as described in detail in Section 6.1).
- The recording starts after a good insonation is obtained.

Contrast agent preparation:

Three alternative contrast preparation methods can be used:

- Saline–Air Mixture: mix 9 mL of isotonic saline with 1 mL of air using a three-way stopcock. The mixture is rapidly exchanged between two syringes at least 10 times to generate MBs
- Saline–Air–Blood Mixture: mix 9 mL of isotonic saline with 1 mL of air and a small amount of the patient’s venous blood (typically 0.5–1 mL). The addition of blood has been reported to increase the sensitivity of the examination by improving microbubble stability and echogenicity (64, 65). The solution is rapidly agitated between two syringes at least 10 times before injection.
- Echovist Preparation: when using Echovist, a suspension of galactose-based microbubbles, prepare according to the manufacturer’s instructions and inject 5 mL per bolus.

In all cases, the contrast agent should be injected as a bolus immediately after preparation in 2–3 s, in order to preserve microbubble integrity and to ensure optimal vascular delivery (65).

Examination procedure:

The first injection is performed under baseline conditions. The software generally allows the operator to mark the exact time of injection for accurate synchronization and subsequent analysis. After the initial administration of the contrast agent, the recording must last at least 30 s and a waiting period of approximately 2 min is recommended before repeating the test.

- If “shower” effects are recorded at basal conditions, the second injection should not be performed. If no shower effect is detected under baseline conditions, the examination is repeated during a VM to enhance sensitivity (or an alternative provocative maneuver). The contrast agent is injected 5 sec before the onset of the VM, which should be maintained for about 10 sec. These events must also be annotated in the recording to facilitate later review and to verify the accuracy of the automatic MB counting.
- The Valsalva maneuver is explained to the patient, and a practice attempt is conducted before the actual examination. The effectiveness of the Valsalva maneuver should be confirmed by observing a reduction of approximately 35% in the mean cerebral blood flow velocity on the Doppler spectrum (66). Even in this case the recording must last at least 30 s.
- When, following two administrations of agitated saline, only a single microbubble signal is observed or the adequacy of the Valsalva maneuver cannot be confidently ascertained, a third, Valsalva-enhanced injection should be undertaken to maximize the diagnostic sensitivity of TCD for the detection of right-to-left shunts.

### 7.1 Final steps

**Figure 3:**
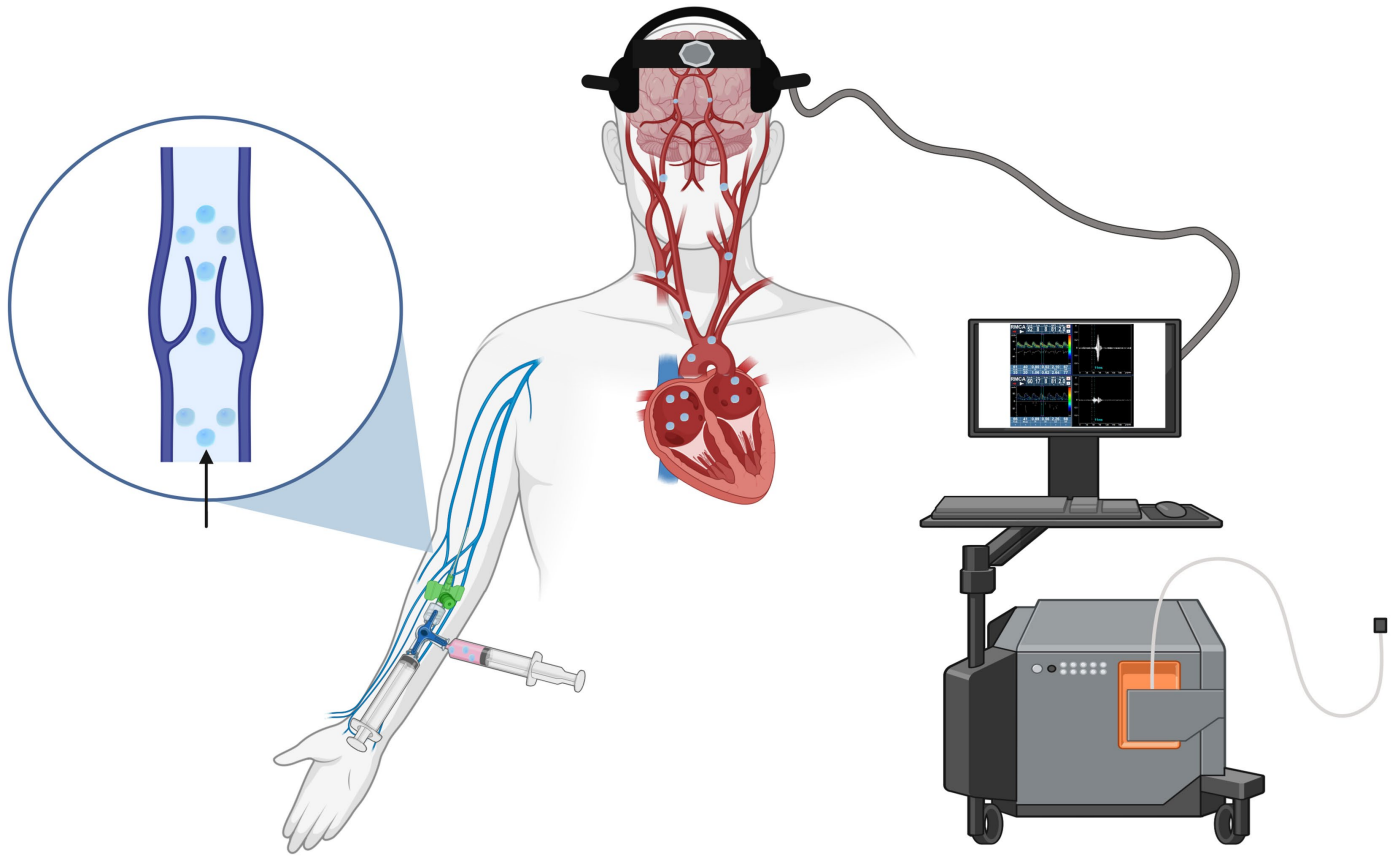
Schematic representation of contrast-enhanced transcranial Doppler (TCD) monitoring for right-to-left shunt detection.

At the end of the examination, standard nursing procedures are carried out for the removal of the intravenous cannula and for the prevention of local bleeding or hematoma formation. The neurosonologist then reviews the study, assessing the recording with visual inspection of each microbubble and manual counting, since the automated system may occasionally double-count a single MB or misclassify a true signal as an artifact. The microemboli detection software generally allows visualization of microbubbles, emboli, or artifacts in three different modes: the Doppler spectrum, the M-mode display, and the raw data analysis. It is this latter modality that enables the assessment of the transient nature of the signal across depths, allowing for an accurate distinction between artifacts and true microbubbles. The examination is subsequently archived on the device memory or, when available, uploaded to the institution’s internal server. (Figure 3 displays the proposed c-TCD protocol, and Table 2 summarizes the main steps).

**Table 2:** Summary of the c-TCD protocol.

Phase	Operational details
Materials and Equipment	TCD with emboli detection software Bilateral probe headset 18-gage needle Isotonic saline or contrast agent Syringes Three-way stopcock
Patient preparation	Supine position 18G needle inserted into the right cubital vein Insonation of at least one MCA using TCD - Instruct the patient for a correct VM
Contrast agent preparation	Mix 9 mL of isotonic saline with 1 mL of air and the patient’s blood If Echovist is used, inject 5 mL per test Agitate the mixture between two syringes using a three-way stopcock ≥10 times to generate MBs Inject the bolus immediately after preparation
Examination procedure	First injection at rest Second injection during VM Inject contrast agent 5 s before VM starts Patient holds VM for 10 s MCA’s peak flow velocity is used to assess VM effectiveness
Final steps	Remove the intravenous cannula Apply pressure at the insertion site to prevent hematoma and bleeding Review of the examination by the operator - Archiving of the examination Report drafting

c-TCD, contrast-enhanced Transcranial Doppler; TCD, Transcranial Doppler; MCA, Middle Cerebral Artery; VM, Valsalva Maneuver; MBs, Microbubbles.

For shunt classification, the criteria proposed by Jauss and Zanette (8) are applied:

- No MBs: negative result
- 1–20 MBs: mild shunt
- >20 MBs without curtain: moderate shunt
- Curtain effect: severe shunt (a dense shower of MBs in which individual bubbles cannot be distinguished).

In cases of unilateral monitoring, a mild shunt is defined by the detection of 1–10 microbubbles, whereas a moderate shunt is identified when more than 10 MBs are observed (8).

### 7.2 Report

The report should include a detailed description of the procedure, highlighting any deviations from the standard protocol. It must contain the raw test results, including the number of MBs detected per artery, both at rest and following the provocative maneuvers, and should conclude with the diagnostic interpretation. It is essential to specify whether arterial monitoring was performed unilaterally or bilaterally, and whether the probes were secured with a headframe or manually held in position during the examination. The type of contrast agent used and the number of injections should be clearly specified, along with details of venous access (gage and anatomical site). The facilitating maneuvers employed must be described in detail, especially if an alternative to the standard VM was used. The report should conclude with the diagnostic summary, specifying the shunt characteristics, permanent, if present in basal conditions or latent, if highlighted after VM, and its grading (mild, moderate, or severe; Table 3).

**Table 3:** c-TCD report components.

Section	Details to include
Procedure description	Note any deviations from standard protocol
Raw test results	Number of MBs detected per artery at rest and after maneuver
Monitoring mode	Unilateral or bilateral MCA monitoring
Probe handling	Held manually or fixed with a headframe
Contrast agent details	Type of contrast agent used Number of injections
Venous access	Gage and anatomical site of venous cannulation
Facilitating maneuver	VM or alternative
Diagnostic Summary	Shunt type: latent or permanent Grading: mild, moderate, or severe

MBs, microbubbles; MCA, Middle Cerebral Artery; VM, Valsalva Maneuver.

## 8 Discussion

Contrast-enhanced transcranial Doppler has progressively established itself as a highly sensitive and non-invasive technique for detecting RLS, particularly in the context of suspected PFO (67). Despite its well-documented diagnostic accuracy, the use of c-TCD in clinical practice remains limited. The principal factor contributing to this underutilization is the steeper learning curve required to master the technique compared with c-TCCD. The acquisition and interpretation of c-TCD signals are inherently operator-dependent, requiring rigorous training and experience to ensure reproducible and reliable results (68). Conversely, c-TCCD leverages ultrasound systems equipped with widely available sector probes commonly used for transthoracic and transesophageal echocardiography, which partially explains its broader diffusion across echocardiographic laboratories. Nonetheless, the relative ease and accessibility of c-TCCD should not be equated with comparable sensitivity or diagnostic reliability. Although preliminary studies have suggested that c-TCCD may demonstrate promising sensitivity and specificity, the paucity of comparative investigations and the lack of standardized protocols preclude its adoption as a validated alternative to c-TCD in RLS diagnosis (69). Indeed, the apparent convenience of c-TCCD risks engendering a false sense of diagnostic confidence, which can potentially lead to false-negative or false-positive findings with significant clinical implications. For this reason, the current evidence does not support replacing c-TCD with c-TCCD in routine practice, and further methodologically robust studies are needed to elucidate its true diagnostic performance. Simplicity of execution must not be conflated with diagnostic rigor. The consistent application of c-TCD within a framework of appropriate clinical indications, robust operator expertise, and standardized methodology remains indispensable to ensure accurate identification of clinically relevant shunts and to avoid the pitfalls of indiscriminate screening (70).

Equally critical is the rigorous selection of appropriate clinical indications for RLS screening.

While PFO is prevalent in approximately 25–30% of the general adult population, its mere anatomical presence does not necessarily warrant diagnostic investigation (71). Incidental findings of a thin interatrial septum, atrial septal aneurysm, or minimal shunt during routine echocardiographic examinations, in the absence of clinical manifestations such as cryptogenic stroke, transient ischemic attack, or other above-mentioned indications, do not constitute sufficient justification for performing c-TCD (13). In such scenarios, the pursuit of further diagnostic testing is unlikely to yield therapeutic consequences and may instead generate unwarranted anxiety and healthcare expenditure. As with any screening modality, the decision to proceed with RLS detection should be based on a clear and evidence-based clinical question, in which the results are expected to significantly impact patient management (72).

Furthermore, beyond operator proficiency and indication selection, attention must be given to procedural aspects that influence diagnostic yield. The supine position provides greater stability for the helmet and facilitates the operator’s work. However, as demonstrated by Lao et al. (63), the upright sitting position does not negatively affect the reliability of the test and can therefore be used when necessary (for example, in elderly patients). Bilateral monitoring of the middle cerebral arteries and the standardized execution of the Valsalva maneuver are fundamental to maximize sensitivity and facilitating accurate shunt quantification (73). Up to 48% of patients require a provocation to elicit a right-to-left shunt and accurately diagnose PFO (74). The VM is considered the standard method for inducing an interatrial pressure gradient and remains the benchmark for other provocative techniques. During echocardiography, it is usually performed by forced expiration against a closed airway, while modified maneuvers such as cough or sniff are also frequently employed. Commonly used provocations include coughing, breath-holding, abdominal or inferior vena cava compression, with the VM being the most widely adopted (75). In specific clinical settings—such as critically ill or mechanically ventilated patients—alternative methods may be necessary when the VM cannot be performed voluntarily. Positive end-expiratory pressure (PEEP) or elevated plateau pressures can increase right atrial pressure and enhance shunt visualization, though these adjustments should not be applied solely for diagnostic purposes. If a patient (such as in aphasic ones) is unable to perform the Valsalva maneuver, abdominal compression or inferior vena cava compression can be used as effective alternatives. The facilitatory maneuvers described for TEE by Thiagaraj et al. can also be successfully applied during transcranial Doppler and transthoracic echocardiography examinations (76, 77).

An additional critical consideration pertains to the strict adherence to established methodological protocols when performing c-TCD examinations. The accuracy and reproducibility of the test are intrinsically linked to the precise execution of each procedural step, including meticulous preparation of the contrast agent, standardized Valsalva maneuver timing and monitoring, and consistent criteria for MB quantification and shunt grading. In particular, the use of bilateral middle cerebral artery insonation with a helmet-mounted probe configuration significantly enhances sensitivity and mitigates the risk of underestimating shunt severity (78). Furthermore, the categorization of results must be rigorously standardized, differentiating between mild, moderate, and severe shunts based on the absolute MBs count and the presence of a curtain effect, while separately evaluating basal and provocation phases (79). Even though modern transcranial Doppler systems incorporate automated embolus detection software, the operator’s interpretative oversight remains essential to reliably distinguish true signals from artifacts (80). Several studies have demonstrated a correlation between the shunt grade detected by c-TCD—using the scale proposed by the International Consensus Conference—and the size of the PFO assessed by TEE (43, 81).

An alternative classification was proposed by Spencer et al., identifying six categories of shunt severity (Grade 0: no MES detected; Grade I: 1–10 MES; Grade II: 11–30 MES; Grade III: 31–100 MES; Grade IV: 101–300 MES; Grade V: >300 MES) (44). The Spencer scale was designed for bilateral monitoring, with MESs detected using power M-mode TCD across multiple contiguous sample gates. Although the original comparative study suggested that this classification improved the predictive value of c-TCD for identifying large PFOs on TEE (82), there is limited supporting evidence, and subsequent validation studies are lacking. Recent literature continues to employ the original International Consensus classification, as also endorsed by the experts consulted for the development of this protocol. To ensure data comparability across centers and between screening and post-closure follow-up studies, a single, standardized grading method must be consistently applied.

Failure to adhere to these methodological requirements can compromise diagnostic validity and potentially lead to misinformed clinical decision-making.

Special attention must also be directed toward peculiar clinical scenarios in which RLS detection assumes a pivotal role. These include cryptogenic stroke occurring in young women during pregnancy or the puerperium, refractory migraine with aura, decompression illness in divers, and professional diving exposure, where the presence of a PFO or other shunt may substantially alter management strategies. In these populations, a comprehensive multidisciplinary assessment involving neurologists, cardiologists, hematologists, and neurosonologists is imperative to ensure accurate diagnosis and appropriate therapeutic planning. The neurosonologist’s expertise in conducting and interpreting c-TCD examinations is particularly indispensable in these contexts, as it cannot be supplanted by the convenience of TCCD performed by third-party operators without formal training in neurosonology (83). Ultimately, the evaluation of right-to-left shunt in such high-stakes scenarios demands rigorous methodology, standardized protocols, and the involvement of specialized personnel to safeguard diagnostic accuracy and optimize patient outcomes.

## 9 Conclusion

In conclusion, the detection of RLS using contrast-enhanced transcranial Doppler ultrasound represents a cornerstone in the diagnostic evaluation of conditions such as cryptogenic stroke and other clinically relevant scenarios associated with patent foramen ovale. Despite its well-established sensitivity and non-invasive nature, the optimal use of c-TCD necessitates rigorous methodological adherence, specialized operator expertise, and judicious patient selection based on clear clinical indications. The proliferation of alternative techniques, such as transcranial color-coded Doppler, while appealing in terms of accessibility and ease of use, remains unsupported by robust comparative evidence and should not be considered equivalent to c-TCD in routine diagnostic workflows. To ensure diagnostic accuracy and to avoid the risks inherent to indiscriminate or technically suboptimal application, standardized protocols encompassing contrast preparation, bilateral monitoring, provocation maneuvers, and microbubble quantification must be consistently implemented. Furthermore, interdisciplinary collaboration among neurosonologists, neurologists, cardiologists, and other relevant specialists is imperative in high-stakes clinical contexts where diagnostic findings may decisively influence therapeutic strategies. This position paper highlights the importance of dedicated training, procedural standardization, and evidence-based use of c-TCD to ensure the quality and reliability of RLS detection, thereby optimizing patient care and clinical outcomes.
