# Supplementary material for: Right-to-left shunt and transcranial Doppler as a diagnostic tool: when and how to run it. Position statement by the Italian society of neurosonology and cerebral haemodynamics
Source: Front Neurol. 2025 Dec 10;16:1668891. doi: 10.3389/fneur.2025.1668891 (PMC12728742; doi:10.3389/fneur.2025.1668891)
Supplement: Supplementary file 1 [file Table_1.docx]

| **Supplementary Table 1** | | | |
| --- | --- | --- | --- |
| **Experts** | **Name of the Center** | **Location** | **Conflict of interests** |
| Baldini Mariella | Neurology Ward, Hospital San Giuseppe | Empoli, Italy | None |
| Bella Rita | Neurology Ward, University Hospital G. Rodolico-San Marco | Catania, italy | None |
| Cenciarelli Silvia | Neurology and Stroke Center | Città di Castello, Italy | None |
| Colon Serena | Neurology Ward, Hospital San Giuseppe | Empoli, Italy | None |
| Cramaro Antonella | Neurosonology Unit, University Hospital Careggi | Florence, Italy | None |
| Diomedi Marina | Comprehensive Stroke Center, Department of Systems Medicine, University of Tor Vergata | Rome, Italy | None |
| Filippi Andria | Neurology Ward, San Luca Hospital | Lucca, Italy | None |
| Marinoni Marinella | Neurosonology Unit, University Hospital Careggi | Florence, Italy | None |
| Mazzucco Sara | Wolfson Centre for Prevention of Stroke and Dementia, Nuffield Department of  Clinical Neurosciences | Oxford, United Kingdom | None |
| Miceli Giuseppe | Internal Medicine with stroke care ward, University Hospital Giaccone | Palermo, Italy | None |
| Sassos David | RCCS-Policlinic Hospital San Martino | Genova, italy | None |
| Trapani Sara | Neurosonology Unit, University Hospital Careggi | Florence, Italy | None |
| Zedde Maria Luisa | Neurology Unit, Stroke Unit, Azienda Unità Sanitaria Locale-IRCCS di Reggio Emilia | Reggio Emilia, Italy | None |
| Zini Andrea | Neurology Ward, Neurologic Sciences Institute of Bologna | Bologna, italy | None |
